# Involvement of people with schizophrenia in decision-making in rural Ethiopia: a qualitative study

**DOI:** 10.1186/s12992-018-0403-4

**Published:** 2018-08-22

**Authors:** Sally Souraya, Charlotte Hanlon, Laura Asher

**Affiliations:** 1 Department of Population Health, Centre for Global Mental Health, London School of Hygiene and Tropical Medicine, London, UK; 2 Health Services and Population Research Department, Centre for Global Mental Health, King’s College London, Institute of Psychiatry, Psychology and Neuroscience, London, UK; 3 Department of Psychiatry, Addis Ababa University, College of Health Sciences, School of Medicine, Addis Ababa, Ethiopia; 4 Division of Epidemiology and Public Health, University of Nottingham, Nottingham, UK

**Keywords:** Decision-making, Patient participation, Community-based rehabilitation, Convention on the rights of persons with disabilities, Schizophrenia, Psychosocial disabilities, Mental health, Community mental health services, Human rights, Ethiopia, Developing countries

## Abstract

**Background:**

The involvement of people with psychosocial disabilities in decision-making is a fundamental component of a person-centred and recovery-oriented model of care, but there has been little investigation of this approach in low- and middle-income countries. The aim of this study was to explore the involvement of people with schizophrenia in decision-making relating to their care in rural Ethiopia.

**Methods:**

A qualitative study was conducted in rural Ethiopia as part of the Rehabilitation Intervention for people with Schizophrenia in Ethiopia (RISE) project, involving two focus group discussions (*n* = 10) with community-based rehabilitation workers, and 18 in-depth interviews with people with schizophrenia, caregivers, health officers, supervisors and a community-based rehabilitation worker. Thematic analysis was used to examine major themes related to involvement in decision-making in this specific setting.

**Results:**

Involvement of people with schizophrenia in decision-making in this rural Ethiopian setting was limited and coercive practices were evident. People with schizophrenia tended to be consulted about their care only when they were considered clinically ‘recovered’. Caregivers typically had a prominent role in decision-making, but they also acquiesced to the views of health care professionals. People with schizophrenia and caregivers were often unable to execute their desired choice due to inaccessible and unaffordable treatment.

**Conclusions:**

Community-based rehabilitation, as a model of care, may give opportunities for involvement of people with schizophrenia in decision-making. In order to increase involvement of people with schizophrenia in rural Ethiopia there needs to be greater empowerment of service users, wider availability of treatment choices and a facilitating policy environment. Further studies are needed to explore concepts of person-centred care and recovery across cultural settings.

**Electronic supplementary material:**

The online version of this article (10.1186/s12992-018-0403-4) contains supplementary material, which is available to authorized users.

## Background

The burden of schizophrenia in Ethiopia manifests in high levels of disability [1] and mortality [2], a heavy burden on caregivers [3], stigma [4, 5] and human rights violations [6]. Many people who require treatment are not able to access it. Thus, the treatment gap is huge, reaching 90% in rural areas [7], with many individuals reliant on family support and traditional treatments [8]. In light of the scarcity of human and financial resources, a challenge exists of how to ensure that services match international recommendations in terms of promoting the use of individualized treatment [9], including the involvement of people with psychosocial disabilities in decision-making relating to their care. The right of people with psychosocial disabilities to make their own choices is a key principle of the United Nations Convention on the Rights of Persons with Disabilities (CRPD) [10], which was ratified by Ethiopia in 2010.

Involvement in decision-making is considered to be a fundamental component of person-centred and recovery-oriented models of care [11]. These models rest on the assumption that care should respect the needs, experiences and rights of the individual with a mental health problem [12, 13]. As such, both models are grounded in the autonomy-focused value systems of the Western countries where they were developed. The cross-cultural applicability of these approaches and the assumptions upon which they rest have been questioned [14].

Shared decision-making is a model that falls between the traditional medical model and the informed choice model [15, 16]. Its practice ensures bidirectional exchange of information between service users and care providers. Consensus is built on the preferred treatment, in a supportive context of shared responsibility, whilst respecting the values and preferences of service-users [15–17]. This differs from the informed choice model, which implies that the care provider only transfers the information to the patient, who will then make the decision alone [15, 16, 18].

Overall, the evidence about the impact of shared decision-making on service users’ outcomes in mental health is limited [19] and inconclusive [20]. However, in high-income countries, interventions designed to involve people with schizophrenia in decisions relating to their treatment have been associated with better outcomes, especially in enhancing long-term medication adherence [21, 22]; reducing re-hospitalisation [23]; improving social functioning [24]; and increasing satisfaction in community-based programs [24]. Moreover, a recent systematic review and meta-analysis demonstrated that shared decision-making in mental health care can lead to the reduced use of compulsory treatment [25]. However, that study did not find a clear effect on the ability to make decisions or the quality of the therapeutic relationship [25].

Concerns have been raised about the capacity of people with schizophrenia to make decisions about their treatment [26] and the impact of negative symptoms on their motivation to be involved in the process [27]. However, there is evidence that people with schizophrenia have an interest in, and are able to participate in, decision-making [28–30], particularly in relation to choice of psychotropic medications [31]. The views of people with schizophrenia on involvement, what drives participation or non-participation and the impact of their preferences on service engagement and outcomes remain under-researched areas [32–34].

Despite the increased interest in involving people with psychosocial disabilities in decision-making, the gaps in knowledge and feasibility mean that this approach is still not widely adopted in either high or low-income country settings [35]. In line with the CRPD principles, the Ethiopian National Mental Health Strategy [36] aims to develop and implement mental health services which respond to the needs and choices of people with psychosocial disabilities and their caregivers; promote recovery and social inclusion; and counter stigma, discrimination, and human rights abuses. Approaches such as person-centred care, education and participation of service users, carers and communities constituted the main values and principles of the strategy. In addition, concepts of “informed decision-making”, “empowerment” and “peer-support groups” were promoted.

The PRIME (PRogramme for Improving Mental healthcarE) programme [37, 38] aims to evaluate the integration of mental health into primary care in five low and middle income countries (LMICs), including Ethiopia, guided by the WHO Mental Health Gap Action Programme (mhGAP) [39, 40]. Whilst the mhGAP intervention guide specifies ‘the right of the person to be involved in every decision that concerns his or her treatment’, it does not provide guidance on how this should be achieved. The Rehabilitation Intervention for people with Schizophrenia in Ethiopia (RISE) [41–44] project is nested within PRIME. In the RISE pilot study and cluster randomised trial, community-based rehabilitation (CBR) [45] was delivered by trained lay workers through home visits covering psycho-education, family intervention, adherence support, and support to return to work and community activities, alongside community mobilization [41, 42]. CBR workers do not administer psychotropic medication themselves, but support participants to access treatment in primary care. The CBR workers training included shared decision-making as a general principle of care.

An understanding of the experiences of involving people with schizophrenia in decision-making in Ethiopia is needed to guide efforts to improve access to care in this country and other resource-constrained settings [44]. The aim of this study was to understand the extent of involvement of people with schizophrenia in decision-making relating to their care in Ethiopia in the context of a CBR programme and to determine the main factors influencing these processes.

## Methods

### Setting

This study was conducted as part of the RISE [41, 42] 12-month pilot study [44] in the Sodo district of Ethiopia, which is also the setting of the PRIME programme [37, 38]. Sodo district is a rural area located 100 km south of Addis Ababa, the capital of Ethiopia, which is characterized by a lack of infrastructure in terms of electricity and sanitation, and a low population literacy level (45%) [46, 47]. There are eight health centres, where health officers and nurses deliver care, and 58 satellite health posts staffed by health extension workers [47, 48]. Most mental health care is provided at the health centre level, with costs being out-of-pocket for the majority of the population. Access to medication for people with schizophrenia is limited to first generation antipsychotics (chlorpromazine and haloperidol) with sporadic access to depot injection. Medications to alleviate side effects (e.g. anticholinergic medication) are rarely available [49]. A psychiatric nurse-run outpatient clinic is available at Butajira, which is 30-50 km from the district with limited paved roads. People with psychosocial disabilities are also referred to or seek inpatient care at the national referral hospital, Amanuel Specialised Mental Hospital, which is located in Addis Ababa and inaccessible to most people. Traditional and religious healers constitute major sources of care for people with psychosocial disabilities as in the rest of Ethiopia. There are various types of traditional healers (herbalists, *tanqway* (‘sorcerer’), and bonesetters). However, the first port of call in the case of severe psychosocial disabilities is typically holy water sites linked to the Orthodox Christian church (27 sites in Sodo), where people drink the holy water or are baptized in it to gain the benefits [48, 50].

### Data collection

Data were collected between January and May 2015, after two or three month’s participation in the 12 month CBR programme. Two focus group discussions (FGDs) with 10 CBR workers and 18 in-depth interviews (IDIs) with six people with schizophrenia, seven caregivers, two health officers, two supervisors and one CBR worker were conducted. The age range of people with schizophrenia who participated in the IDIs was 18 and 70 years old and four of the six participants were male. All IDIs and FGDs were based on topic guides with open-ended questions focused on the acceptability, feasibility and impact of the RISE CBR program, including a set of questions on involvement in decision-making. The core questions were: Who is involved in decision-making about mental healthcare? How are the decisions made? What are the challenges to involving people with schizophrenia and caregivers in this process?

Data collection was conducted in Amharic by an Ethiopian research assistant with a Masters level degree and experience in qualitative research. IDIs and FGDs were audio-taped, transcribed verbatim, and then translated into English. A member checking meeting [51] was held with one supervisor and ten CBR workers to validate the primary analysis and ensure that it accurately reflected their viewpoints. The member checking was facilitated in English by LA and SS, with translation by the research assistant.

### Data analysis

Data management and coding were conducted using NVivo 10.2.1 [52, 53]. Transcripts were coded independently by LA and SS, and discussed together with the research assistant and CH to ensure reliability, internal coherence and consistency of the coding framework, as well as capturing the cultural context. Thematic analysis was used to examine major themes related to involvement in decision-making [54]. However, a recursive process constantly moving back and forth between the entire data set and the notes from the member checking allowed existing themes to be emphasized and additional sub-themes to emerge, such as “capacity”. The accounts of the different categories of participants involved in this study (e.g. people with schizophrenia, caregivers, CBR workers) were also compared in relation to the themes emerging from the data. Quotations from participants were embedded within the analytic narrative to illustrate and support the analysis.

### Ethics approval and consent to participate

This study received ethical approval from the LSHTM MSc Research Ethics Committee [Ref.10163], the LSTHM Interventions Research Ethics Committee [Ref.7035] and the Addis Ababa University College of Health Sciences Institutional Review Board [Ref.083/13/Psy]. Written informed consent was obtained from all participants. Participants who were non-literate gave a thumbprint and a witness signed to confirm that the study had been explained according to the written information leaflet. All data were de-identified to ensure anonymity of the participants.

## Results

Three major themes were identified: how decisions are made about care; what factors affect involvement; and what influences the choices made.

### How decisions are made about care

#### Communication and role of CBR workers

Most CBR workers and supervisors described how they advise people with schizophrenia on the available treatments, their advantages and side effects (Additional file 1). Asking people with schizophrenia what their goals are, listening to their needs and respecting their opinions constituted fundamental elements of this process. CBR workers were then indirectly involved in supporting treatment choices to be enacted, for example by assisting individuals to attend the health centre. A supervisor described how they were increasingly involving both people with schizophrenia and caregivers:

*“Most of the time, the patients give a special place for those who give them attention and give them advice. I think this is what they are deprived of.… we give them an opportunity to develop an interest in talking about their issues. [Before] When we ask some questions about something, which is an issue for the patient, the caregiver will answer and we will leave the patient without asking that question. This should not be the way. Therefore what we do is we ask the individual as well as the caregiver. We are now asking both about the issue, which concerns them equally. We are also asking questions which are for the patient only”*. (Supervisor, IDI07)

Using clear simple information and giving more time for people with schizophrenia to understand their options and express their choices were underlined as effective strategies to actively engage them in the decision-making process. One CBR worker explained:

*“[The family] don’t give [the person with schizophrenia] time; I wait for her calmly to respond and after some time she answers. Then, I show and explain to them that she answers like this by taking her time and she should practice like this slowly…”* (CBR worker, IDI20)

#### Adjustments to treatment plans

Adjustments to treatment plans were sometimes made as a result of people with schizophrenia actively requesting changes; or by being asked about their preferences at the health centre or in CBR sessions. Several CBR workers and health officers emphasized that the requests of people with schizophrenia are taken into account not solely to enhance their involvement in decision-making, but to equally address issues of non-adherence to treatment and avoid refusals of care. Health officers changed treatments from oral medication to injection and CBR workers adjusted the timing and frequency of CBR sessions, according to the needs of people with schizophrenia. These were reported as the main mechanisms indicating ‘involvement’ according to CBR workers and health officers in this study.

#### Prominence of caregivers’ involvement

The caregivers’ involvement in decision-making often took precedence over that of people with schizophrenia. This prominent role in decision-making was linked to caregivers’ crucial role in supporting recovery. Caregivers’ collaboration with health care providers was reported to be essential to ensure service users’ access and adherence to treatments. A health officer explained:

*“Even if we say it is the patient who is suffering, the responsibility of giving support and care lies on the caregivers. If the caregiver didn’t participate in the decision making process, the treatment will not give the expected results. The caregiver is the one who gives the medicine to the patient. It is also the caregiver who can decide on the frequency of visit and can inform on the changes observed in the patient. The caregivers are especially important in cases where the patient doesn’t give any response...”.* (Health officer, IDI18)

Furthermore, caregivers were perceived to be more active in the decision-making process than people with schizophrenia, for example by asking questions and expressing their ideas. Thus, health officers, supervisors and some CBR workers expressed a preference for the involvement of caregivers. From the perspective of people with schizophrenia, the involvement of their caregivers was considered in some cases as an expression of their desire to care for the person. Thus, respondents reported trusting caregiver to make decisions on their behalf. Moreover, caregivers were considered to have the right to be involved and make decisions as they are equally affected by the burden of the illness on them. A person with schizophrenia described how his son took him to the hospital as he was concerned and stressed:

*“It was my son who took me there [the hospital]. He was too concerned about me. He is the one who loves me a lot. When he became too stressed about my situation, people told him to take me to Amanuel hospital.”* (Person with schizophrenia, IDI02)

Yet in some cases, caregivers seemed to completely exclude their relatives and make decisions on their behalf without even informing them. One CBR worker explained:

*“They [her daughter and son] don’t even tell the patient what they planned to do for her care. They were entitled with the decision making of everything in her life. They don’t consult or ask her…Even in relation to going to church they fear taking her as she might disturb there. She is restricted from all those freedoms.”* (CBR worker, P3-FGD1)

A person with schizophrenia expressed his frustration of not being informed or having the choice in relation to his care:

*“If I knew [we were going to holy water] I wouldn’t go, but they [his mother and another person] decided. I don’t like that! … They would take me either by force or politely”.* (Person with schizophrenia, IDI17)

#### Coercive care

Facing relapse due to the refusal of care and non-adherence to treatment, some caregivers adopted coercive approaches. These included scaring service users to influence their treatment choices; mixing medication into food without telling the person with schizophrenia; and physical restraint. There was a consensus among health officers, CBR workers and supervisors that coercive approaches are ethically inappropriate. However, they all acknowledged the usefulness of such approaches to guarantee that the person is taking medication. Thus, they argued that the use of coercion as a last resort, after exhausting all other alternatives, was justified as long as it was not harmful and used for the benefits of the service users (Additional file 1). Health officers supported their views by explaining how some service users, who were given medication without their consent, later recovered and took the medicine by themselves:

*“There are cases where the medication is given with food and injection medicines are administered by use of force…without the patients will. The patients will thank you when they recover and stabilize. I know patients who were treated like that and have returned to their normal life and work. This shows that we might not get this result if we had waited for the patients consent and tried to understand their feelings…”.* (Health officer, IDI16)

A divergent view from one caregiver favoured persuasion over the use of force to convince people with schizophrenia to follow treatment:

*“Yes, the use of force doesn’t work at all. There is nothing could be done with force. He is not cooperative. It is helpful to convince him and let himself decide. I think that is the better way…though he might not be willing at the beginning, at last he will be convinced…but, it is after a series of discussions and arguments...”.* (Caregiver, IDI01)

Additionally, one supervisor explained how CBR could play a role in creating awareness among caregivers on how to respect the dignity and ensure the safety of the person with schizophrenia when the use of coercion, such as physical restraint, is needed.

### What affects involvement in decision-making

A number of individual factors (capacity, intellectual disability, motivation and financial capacity) and service delivery factors (setting and CBR worker fear of failure) were identified as affecting involvement in decision-making.

#### Individual factors

#### Capacity

Perceived mental capacity to make decisions emerged as an important factor that determined whether attempts were made to involve people with schizophrenia in decision-making. Views varied across the respondents on whether having schizophrenia per se necessarily indicated lack of mental capacity. However, health officers tended to consider schizophrenia as synonymous with incapacity. There was a strong belief that illness severity impacted on the capacity of people with schizophrenia to be involved. Symptoms such as confused thoughts, delusions and lack of insight were considered to hinder people with schizophrenia from participating effectively in decision-making.

*“As the illness becomes severe, even when [the person with schizophrenia] is asked about decision-making, what she says is outside of the issue we want to decide about. She simply talks on her own. We couldn’t understand what she says as her talk is not normal. I think that this might influence the judgment that she has no capacity to decide”.* (CBR worker, P3-FGD1)

Health officers, CBR workers and supervisors suggested that capacity is enhanced after receiving treatment and recovering, enabling people with schizophrenia to participate more actively and make decisions that they viewed as ‘better’. People with schizophrenia who had recovered clinically were reportedly given more opportunities to be involved in decision-making. Instead of imposing opinions on them, respondents reported that they often asked them what they wanted and listened to how they would prefer to be helped. In recovery, the choices of the person were considered to be trustworthy and thus more respected (Additional file 1).

Nevertheless, attitudes of caregivers towards the capacity of people with schizophrenia were not consistent with those claimed by health officers. In some cases, caregivers’ attitudes appeared not to change over time; irrespective of the person’s recovery. The person with schizophrenia appeared to be stigmatized and their capacity was judged based on previous situations when their symptoms were worse (Additional file 1). Equally, the type of decisions made by people with schizophrenia seems to influence the judgment of their capacity not only by caregivers but also by CBR workers. It was implied that ‘better’ decisions involved adherence to medication and compliance with health professionals’ advice. CBR workers underlined how the concerns of people with schizophrenia about their medications and/or a decision not to attend treatment are sometimes dismissed as signs of relapse and considered as irrational and reflective of lack of capacity.

In addition, the limited abilities of the two CBR participants with comorbid intellectual disability to understand and communicate were seen as a major obstacle towards their involvement in decision-making. Thus, in these cases, caregivers, CBR workers and health officers tended to make decisions on behalf of the person. One CBR worker explained:

*“As she also has intellectual disability, she is not conscious about her overall situation. Therefore, she couldn’t decide by her self. We are the ones who decide on behalf of her. We are deciding whether she has to go to Holy water or health centre instead of her. It is because she doesn’t say anything, it is only her family who decides with us”.* (CBR worker, P5-FGD2)

#### Motivation and expectations

Motivation and expectations of people with schizophrenia and caregivers also appeared to affect the extent of their involvement. One CBR worker reported that people with schizophrenia tend to keep their opinions to themselves and lack motivation to be involved in decision-making due to pervasive stigma. A health officer highlighted how it is difficult to engage people who are not motivated and willing to express themselves. They also underscored how unmet expectations of care can affect the judgement of people with schizophrenia and their caregivers to make the “right” treatment decision:

*“If the treatment didn’t bring the result they [the person with schizophrenia and the caregiver] wanted; and the illness became worse, the patient might try to escape or think you have misled him and try to attack you, because he spent a lot of money and didn’t see any change. This will create prejudices for the caregiver and the patient and they will not be able to make the right decision”.* (Health officer, IDI18)

#### Age and position in the family

The age and position of people with schizophrenia and caregivers in the family was discussed as influential on their perceived decision-making ability. One supervisor explained that from the perspective of the family, a father, who is responsible for the family, would be given the opportunity to make his own decisions. In contrast a son or daughter living with their parents might have less power within the family and therefore not be considered capable of making decisions.

#### Financial capacity and power

The financial capacity of people with schizophrenia and caregivers was discussed as an important factor in determining who is involved in decision-making. Household finances were typically in the hands of caregivers; therefore they held the power to make decisions regarding treatment. When families were under financial constraints, other family or community members were sometimes involved and influenced on the decisions made to access treatment.

*“At the beginning, it was me [the caregiver] who decided, as the Holy water doesn’t require any money, so I could take her [the person with schizophrenia] there easily. However, for the later [treatment at the health centre], it was him [her nephew] who decided, as I don’t have money”.* (Caregiver, IDI03)

CBR workers highlighted some exceptions where people with schizophrenia decided independently about their treatment and bought medication with their own money. These were people who were working and had gained back their productivity and financial independence.

#### Service delivery factors

#### Setting

The setting where decision-making takes place was considered to affect the feasibility and acceptability of involvement. The high staff workload at health centres was discussed as an obstacle. However, the greater time capacity of CBR workers and the provision of home-visits were perceived to enhance the participation of people with schizophrenia and caregivers. One CBR worker described how home visits increase confidentiality; and thus, they offer people with schizophrenia the possibility to interact more freely in the decision-making process:

*“People with schizophrenia and their caregivers want confidentiality about their illness. Through home visits, they could feel free to express their views. They could express their opinions and concerns freely as there is only them and us there”.* (CBR worker, P2-FGD2)

Moreover, the support received during home visits was greatly appreciated and accepted by people with schizophrenia and caregivers, who felt engaged in these visits especially because the timing and frequency were tailored to their convenience (Additional file 1).

#### CBR workers fear of failure

Fear of failure due to refusal of care by people with schizophrenia was indicated as a major concern for some CBR workers. They described the stressful and demoralizing feeling associated with a person’s lack of improvement and that refusal of care negatively influenced their motivation and relationships with people with schizophrenia (Additional file 1). Thus, some CBR workers adopted a persuasive approach to convince participants to continue treatment and CBR by re-emphasising the benefits and potential impact on their lives. In some cases, CBR workers mustered the support of caregivers and health officers in both evaluating the risks and stressing the benefits of treatment and CBR sessions, based on their insights into the person’s situation and care plan.

### What influences on the choices made

#### Clinical recovery

There was a strong belief among CBR workers that symptomatic improvement affects the way people with schizophrenia perceive their need to continue the care. They explained how people with schizophrenia started to refuse care including CBR, medication, and attending the health centre, once they began feeling better. For some people, these treatments were considered as options to be sought only in case of relapse or severe illness. Lack of improvement might equally affect the motivation of people with schizophrenia and caregivers to continue the treatment. A health officer explained:

*“People get bored when they don’t see changes…The treatment of mental illness needs long follow up. You might not see changes sometimes. This is a big challenge in the treatment of the illness…Some patients recover from their illness. Some do not recover. The families of the patients who haven’t recovered have a tendency to be demoralized. The caregivers may bring the patient from remote areas; villages might not have access to transport. In addition, the cost of transport and treatment is very high…”.* (Health officer, IDI18)

#### Side effects of medication

Despite recognising the importance of medication in treating their symptoms, the side effects of anti-psychotic medication remained a major concern, which frequently led to non-adherence. People with schizophrenia and caregivers were often concerned about sedating side effects affecting their daily life and productivity, in particular the ability to do manual labour, whilst one family were concerned the side effects might prove to be life threatening. From their side, CBR workers and health officers underlined their responsibility to clearly explain the side effects of medication to people with schizophrenia and caregivers.

*“…As the medication created fatigue on her situation they suspect that this might lead to death. They stopped the medication and went to other treatment options because of that. However, after trying other options, they returned back [to medication]”.* (CBR Worker, P3 FGD1)

#### Poverty and lack of access to free medicine

In the absence of free medication, lack of financial capacity to pay for medication was discussed by all participants in this study as a major obstacle, which often guided decisions relating to the care of people with schizophrenia.

*“The patient whom I am working with is from a poor family. The medication prescribed for him needs one hundred forty five birr [approximately US$ 5]. …. What I want to add here is about the challenge which the caregiver is facing and even she was on the way to decide to stop [the medication] because of the economic challenge. She even asked me to find support for her as she couldn’t be able to help her children. The father of the patient has nothing to support her*”. (CBR worker, P4 FGD2)

#### Relationship with health professionals

In the context of this study, there appeared to be a power dynamic, in which the knowledge and opinions of health professionals (health officers and CBR workers) were usually considered valuable and trustworthy. In some cases, even when asked about their preferences and given the choice to decide, people with schizophrenia and caregivers seemed to trust the opinions of health professionals and delegate them to make the decisions. Good communication, attention and respect towards the person with schizophrenia were identified to play an important role in enhancing this confidence.

*“All the health professionals have a good attitude towards [the person with schizophrenia]. Especially [xxxx], … at the health centre, gives him good attention. [The health officers] ask him his preference whether it is better to take medication or injection, while he tells them that he is okay with both options. When they ask him to have an injection, he takes the injection without any resistance. He has no problem in this respect. He tells him that he could take medication if they think that is appropriate or if they think injection is appropriate respectively”* (Caregiver, IDI10)

#### Traditional treatments

Decisions to use traditional treatments appeared to be most often driven by the beliefs of people with schizophrenia and caregivers and also influenced by relatives, the local community and religious leaders.

*“It was both of us [caregiver and person with schizophrenia] together [who decided to go to Holy water]. Other people also recommended the Holy water. So, It was all of us who decided with the people who has seen his situation...as it might be an evil spirit…It was with the assumption of treating the illness with Holy water. I took him after a discussion. Then, he attended the Holy water treatment for some days”.* (Caregiver, IDI01)

Nevertheless, there were some divergent opinions regarding traditional treatments among people with schizophrenia and caregivers, who refuse to seek these types of treatments, despite them being recommended by others.

*“I couldn’t go to such places [Traditional treatments]. I can’t attend this “Chida” [traditional] treatment. I believe in one God who created the earth. I worship only one God not any other… I haven’t tried any Holy water. I don’t want to go there although they [his family] asked me to”.* (Person with schizophrenia, IDI08)

## Discussion

This study shows that in practice there is limited operationalization of involvement of people with schizophrenia in decision making in this rural Ethiopian setting. Coercive practices are not uncommon. A CBR programme may give service users more opportunities for involvement compared to a primary care setting. However in general people with schizophrenia tend to be consulted about their care only when they are considered clinically ‘recovered’ and in the absence of comorbid intellectual disability. These practices may reflect a pervasive stigma towards people with mental illness. Caregivers typically have a more prominent role in decision-making, but even they often acquiesce to the views of health care professionals. People with schizophrenia and caregivers are often unable to execute their desired choice due to inaccessible and unaffordable treatment.

### Strengths and limitations

This is among the first studies to explore the involvement of people with schizophrenia in decision-making relating to their care in a low-income country setting. Comparing the perspectives of people with schizophrenia, caregivers and service providers allowed a holistic and comprehensive understanding of current involvement, and enabled us to identify the roles of each stakeholder in the process. The study also sought to understand involvement across the spectrum of functioning, by including caregivers and CBR workers of people with schizophrenia who were included in the pilot, but could not participate in the interviews due to cognitive impairment. In addition, conducting member-checking increased the validity of the findings.

However, there are limitations to this study. First, this was an exploratory study conducted in the specific setting of a pilot study of CBR. The findings may not be generalisable to other settings where CBR does not exist. Furthermore, data on decision-making were collected at one time point early in the RISE pilot study so do not allow understanding of how perceptions may have changed over time during the course of treatment or CBR. The broader RISE pilot evaluation, which drew on qualitative and process data over 12 months, also identified excessive persuasion to take medication amongst a minority of CBR workers. Second, information bias may have occurred due to CBR workers and supervisors emphasizing their efforts to involve people with schizophrenia and caregivers in compliance with the RISE study protocol. The intimate involvement of one of this study’s authors in the RISE project might also have led to a more favourable depiction by the CBR workers. Equally, despite the anonymity of the interviews, people with schizophrenia and caregivers might have been reluctant to criticize CBR workers and health officers for fear of damaging their relationships with them. Furthermore, socio-cultural norms and the financial dependency from caregivers may have potentially influenced the frankness with which people with schizophrenia discussed their relationship with caregivers. An observational approach could have reduced this response bias. Third, despite triangulating the data, the analysis was mostly shaped by opinions of CBR workers, supervisors and health officers, which were generally more clearly articulated than those of people with schizophrenia and caregivers. Nonetheless, even in this context which would be expected to lead to more favourable depictions of service user involvement, the extent of involvement in shared decision-making appeared to be limited.

### Comparison of findings

In this study only limited evidence of shared decision-making [15, 55] was identified. This supports findings from a previous qualitative study in rural Ethiopia, which showed that people with psychosocial disabilities and caregivers are often poorly informed about their rights, the illness, available treatments and their risks and benefits [56, 57]. Moreover, the decision-making model identified in our study was in some cases dominated by caregivers and health professionals. Discussions regarding treatments tend to be characterized by asymmetric interactions, where health officers and CBR workers seemed to control the exchange of information. However, this needs to be understood within the context of Ethiopian society, where health professionals are trusted and are expected, as in many cultures, to assume an authoritative and prescriptive role [14]. Furthermore, both service users and caregivers in rural Ethiopia may fear expressing their opinions if they include criticism of mental health providers, as this could jeopardize their access to care [57]. Mayston et al. underlined that the extent to which people with psychosocial disabilities in this setting express their opinions is likely limited by their marginalized role in their community [58]. Women in Ethiopia also have low status and have fewer rights than men (for example, less control over household decisions and lower access to education and employment) [59–62]. Thus, it is conceivable that gender norms might constrain the involvement of females, whether it is the person with schizophrenia and/or the caregivers, in decision-making. In one case decision-making power apparently shifted to the nephew of a woman with schizophrenia because her mother was unable to pay for treatment. However, there was insufficient data from our study to draw firm conclusions on the role of gender.

In common with other LMICs, caregivers in this study were often the main decision-makers, rather than a contributor to the process [56]. It has been proposed that in Ethiopian society the rights of families are not separate from those of people with mental illness and that the ‘smallest autonomous unit’ is the family rather than the individual [63]. Thus a collective approach to decision-making is dominant [56], though in our study this did not typically extend to the wider community. In settings where there is limited access to care, caregivers may be forced to over-ride the autonomy of the person with psychosocial disabilities to ensure that they receive effective treatment and that other people are protected [56]. The potential for these relationships to become abusive has been noted [56]. However, in our study, caregivers framed their actions as taking moral responsibility for their family member: in such a context, to give the person freedom to decide whether to take treatment or not might be perceived as irresponsible and even unethical. Similarly, the physical restraint of people with schizophrenia by family members has been conceptualized as a form of care pragmatically employed to protect individuals and the wider community [6]. The tensions between respecting autonomy and ensuring protection from harm equally exist in high-income countries. Yet in rural Ethiopia the dominant mode of personhood is arguably socio-centric, where value is given to reliance on one another and mutual responsibility [14, 64, 65], in contrast to conceptions of disability in Western cultures, which emphasize autonomy and independence. The prominence of caregivers’ involvement in our study was also attributed to the heavy caring and financial burden. It has previously been noted that people with mental illness would not usually seek treatment without the family’s backing [63] and if the family refuses to pay, then the person would often not have access to treatment [56].

It has been proposed that decision-making capacity is considered an ‘all or nothing’ phenomenon in many African countries [66]. People with mental illness, who are found to be lacking capacity, are deprived of their rights and meaningful involvement in the management of their lives on the basis of minimal evidence and their capacity is re-assessed rarely if at all by health professionals [66]. Concerns about mental capacity are often highlighted as a major barrier to individuals’ involvement in decision making [26] and this was a key finding in our study. The CRPD indicates that all persons with disabilities, even those who lack mental capacity, should receive care only on the basis of free and informed consent, and should be offered support to reach autonomous decisions [10]. Article 12 of the CRPD [10] indicates that persons with disabilities have the right to exercise legal capacity according to their own will and preferences at all times [67]. However, it has been argued that the inability of health professionals to override personal autonomy in any circumstances undermines critical rights for people with psychosocial disabilities, including the enjoyment of the highest attainable standard of health [68]. Freeman et al. suggest that the likelihood of recovery, and the resumption of capacity to make treatment decisions in an informed manner, is often diminished without treatment [68]. This reasoning is supported by our study, which found that people with schizophrenia who were clinically recovered were reportedly offered more opportunities for involvement.

Studies from high-income countries, where treatment is widely accessible, have demonstrated that a high proportion of people with schizophrenia are competent to make decisions in relation to their care [69, 70], and illness severity does not necessarily influence participation [71]. However, in our study in which treatment uptake was variable, stage and severity of the illness and comorbid intellectual disability were highlighted as factors associated with low decision-making capacities. Bearing in mind the impact negative symptoms are believed to have on motivation and engagement in the decision-making process [27], findings from our study supported the use of individualized approaches by CBR workers, for example involving service users by giving them more time to express themselves [71].

In this study, involvement of people with schizophrenia was often undermined by caregivers and health professionals focusing on the decision made, rather than the entire process [72]. The legitimacy of decisions not to adhere to medication or to refuse care was often questioned, in light of a perceived lack of capacity. This is consistent with studies from the US reporting that non-adherence is often perceived to be symptomatic of the illness, rather than indicative of patient preferences [73], and a sign of incompetence [74]. Finally, our findings align with recent research in rural Ethiopia, which identified poverty and intolerable side effects of anti-psychotic medication as key reasons for disengagement with mental healthcare [75].

### Implications

The overall findings of this study suggest that the nature and setting of CBR interventions and the role of CBR workers hold substantial promise in enhancing the participation of people with schizophrenia and caregivers in decision-making by facilitating their understanding and encouraging them to express their opinions. However, the feasibility of involvement remains challenging in Ethiopia. Poverty, lack of affordable medications and access to psychosocial care means that real choices for treatment in the context of biomedical care are limited. This hinders the process of decision-making and can lead people with schizophrenia and caregivers to reject care. Thus, the over-riding priority in LMIC is to expand access to care so that people with schizophrenia and caregivers have meaningful choices at an earlier stage in the illness [56]. Such choices might include access to a wider range of psychosocial interventions and anti-psychotic medications with a more acceptable side effect profile.

The establishment of community-based mental health services is a major focus of the Ethiopian National Mental Health Strategy [36] but implementation is patchy due to workforce and funding constraints. CBR has historically been delivered by NGOs in Ethiopia and at present this is the most feasible route for wider implementation.

Once accessing care, people with schizophrenia should be actively involved by being informed of their treatments options and given the right to choose their preferred treatments. This may include on one hand reassurance that care provision will not be affected by expressing an opinion or preference about treatment. On the other hand, it should also include orientation to the notion that people with psychosocial disabilities can still have capacity even if their decision does not accord with health workers recommendations.

Guidance on how to involve people with schizophrenia in decision-making should be included in the mhGAP Implementation Guide, and implemented as part of wider efforts to scale up mental healthcare in primary care in LMIC. Such guidance should be contextualised and acknowledge variations in decision-making norms and values across cultures, and mental health systems, especially where choices are very limited and may be influenced by age, gender, position in the family and socio-economic status. Specific guidance on decision-making involvement in times of crisis may be valuable.

In Ethiopia, the National Mental Health Strategy needs to be supported by mental health legislation and context-specific policies and procedures on implementing, monitoring and evaluating the involvement and addressing stigma and discrimination of people with psychosocial disabilities in inpatient and outpatient care. Legislation should guide capacity assessment and informed consent and should be designed to protect people with psychosocial disabilities who are not consenting to treatment [76]. Legislation could also guide the use of advance directives, which document a person’s preferences for treatment should they lose the capacity to make decisions in the future. However implementation of such approaches may be challenging in the absence of specialist input and appropriate oversight mechanisms.

The empowerment of service users, for example through self-advocacy organisations, may support a broader shifter towards people with psychosocial disabilities being aware of their rights [58], and also contribute to their involvement in strengthening the mental health system [57] .

Cultural, social and religious values remain key to individual decision-making, exemplified by the prominent role of caregivers and the use of religious and traditional treatments. These values need be acknowledged and respected. Increased involvement in decision-making by the individual should not result in the exclusion of caregivers from the process. Indeed, removing caregivers and families from decision-making may amount to the imposition of Western values and is likely to be both unacceptable and unfeasible in the Ethiopian setting. Thus, health professionals should focus on how to orient this involvement to be for the benefit of the person with schizophrenia, who is usually dependent on caregiver support. The process should aim to support the balance between protection from harm and preservation of autonomy [77].

Further studies are needed in LMIC to explore how concepts of person-centred care and recovery as well as the process of involvement in decision-making could be contextualised and adapted to ensure local validity and acceptability across cultural settings [13, 14] and in under-resourced health systems. Of particular interest is how decision-making processes function in settings in Ethiopia and elsewhere where CBR does not exist, and where involvement may be less feasible due to the high workload of care providers and limited emphasis on empowering service users. Future research could also explore how involvement in decision-making can be measured in LMIC, using self-report by service users [78] or as part of broader assessment of healthcare worker competence [79]. The RISE pilot evaluation identified involvement in decision-making as a potential intermediate outcome in the pathway to improved functioning. Future evaluations could further explore the impact of shared decision making on outcomes such as treatment engagement and adherence, functioning and personal recovery. Equally, there is a need to define the mechanisms through which involvement in decision-making could go beyond individual-level care to wider involvement in the system, where people with schizophrenia would be more represented in the planning and delivery of services and their role might be transformed into peer support, empowerment and advocacy [80, 81].

## Conclusion

In this study setting in rural Ethiopia, involvement of people with schizophrenia in decision-making about their care was limited and challenging. Caregivers and health care professionals have prominent roles in the process compared to people with schizophrenia. Decision-making was often hindered by absence of real choices and care was sometimes rejected due to poverty and lack of affordable and accessible medications. In this context, CBR represents a promising model of care that may facilitate and enhance involvement and participation of people with schizophrenia in decision-making about their care. This involvement needs to be supported by greater empowerment of service users, wider availability of treatment choices and a facilitating policy environment.

## Additional file

Additional file 1: Supplementary materials_Quotes. Word document. Table of supplementary quotes. (DOCX 46 kb)

## Abbreviations

CBR: Community-based rehabilitation
CRPD: Convention on the Rights of Persons with Disabilities
FGD: Focus groups discussion
IDI: In-depth interview
LMIC: Low and middle-income countries
mhGap: Mental Health Gap Action Programme
PRIME: PRogramme for Improving Mental healthcarE
RISE: Rehabilitation Intervention for people with Schizophrenia in Ethiopia
