# Supplementary material for: Involvement of people with schizophrenia in decision-making in rural Ethiopia: a qualitative study
Source: Global Health. 2018 Aug 22;14:85. doi: 10.1186/s12992-018-0403-4 (PMC6103856; doi:10.1186/s12992-018-0403-4)
Supplement: Supplementary file 1 — Supplementary materials_Quotes. Word document. Table of supplementary quotes. (DOCX 46 kb) [file 12992_2018_403_MOESM1_ESM.docx]

Table of supplementary quotes

| Themes | Sub-themes | Supplementary Quotes | Participants |
| --- | --- | --- | --- |
| How decisions are made about care | Communication and role of CBR workers | “Most of the time, the patients give a special place for those who give them attention and gives them advice. I think this is what they are deprived of. They haven’t got ever a person who listen to them and give them a solution. Now the CBR workers and also we as CBR supervisors listen to them with attention…We give them a straight-forward solution to their problem. Therefore… we give them an opportunity to develop an interest in talking about their issues. When we ask some questions about something, which is an issue for the patient, the caregiver will answer and we will leave the patient without asking that question. This should not be the way. Therefore what we do is we ask the individual as well as the caregiver. We are now asking both about the issue, which concerns them equally. We are also asking questions which are for the patient only”. | Supervisor, IDI07 |
| How decisions are made about care | Coercive care | “Everyone should be supported. The human right for people with mental illness has high priority… Even if they have to be chained, we [CBR workers] will show them [the caregivers] how the patients should be chained. They could be chained in a way that keeps their safety… There will be a better change as they know what to do in those situations through CBR”. | Supervisor, IDI06 |
| What affects involvement in decision-making | Individual factors / Capacity | “…If the patient is in a better situation, he could decide on any issue related to his life. He could ask what he wants to know. He could even put forward his preferences. He could even justify where to go for what problem. He could tell the family about his experience with his treatment in previous times. … The patients who are not in a better situation simply accept what the caregivers or we told them...” | Supervisor, IDI07 |
| What affects involvement in decision-making | Individual factors / Capacity | “At the start the patients’ decision-making potential was so limited because of the severity of the illness. During follow-ups, many patients have decision-making potential. We give explanations to some of them and the right to choose the type of treatment they like. But we are not doing that always because there are some who lack the potential to make decision. We work by discussing with their families. If we think that the patient has a decision-making potential we give priority to the patient’s choice of treatment. Especially when their health is improving, more decision will be given to patients” | Health officer, IDI16 |
| What affects involvement in decision-making | Individual factors / Capacity | “In relation to decision making, what we faced was the attitudes of the caregivers and family. They consider the patients incapable of doing anything and they consider them as worthless, they know the patient when they act towards them in a bad way but when they become well they don’t consider them as having any decision making and even they don’t give them permission that they can do anything, even when they grow or they don’t involve them in the process of …They don’t give them much attention on what they do and they contribute. this might impact them and feel negatively of them“. | CBR worker - Member-checking |
| What affects involvement in decision-making | Service delivery factors / Setting | “I think the idea [of home visits] is good. It is better to be visited here [at home] instead of going there [to the health centre]… I accept it here and I’m happy with that and he [the person with schizophrenia] also accepts it with full interest. …. [The CBR worker] even asked which time and date is convenient to him and we told her that every two weeks is convenient to us”. | Caregiver, IDI 09 |
| What affects involvement in decision-making | Service delivery factors / CBR workers fear of failure | “Sometimes the CBR workers become disturbed as they face challenges every week. They think that their [people with schizophrenia’s] wellbeing is their responsibility. Then they feel stressed and angry with that. Even there was a CBR worker who requested a patient to be changed. This happened, as the patient was not improving well while other’s patients were improving. Therefore he thought as if his value in the work might be reduced as a result of that…” | Supervisor, IDI06 |
